# Quantum-based modeling implies that bidentate Arg^89^-substrate binding enhances serine/threonine protein phosphatase-2A(PPP2R5D/PPP2R1A/PPP2CA)-mediated dephosphorylation

**DOI:** 10.3389/fcell.2023.1141804

**Published:** 2023-06-12

**Authors:** E. Alan Salter, Andrzej Wierzbicki, Richard E. Honkanen, Mark R. Swingle

**Affiliations:** ^1^ Department of Chemistry, University of South Alabama, Mobile, AL, United States; ^2^ Department of Biochemistry and Molecular Biology, College of Medicine, University of South Alabama, Mobile, AL, United States

**Keywords:** PPP2R5D, PP2A, protein dephosphorylation, ONIOM calculations, enzyme catalysis, phosphoester hydrolysis, transition state

## Abstract

PP2A-serine/threonine protein phosphatases function as heterotrimeric holoenzymes, composed of a common scaffold (A-subunit encoded by PPP2R1A/PPP2R1B), a common catalytic (C-subunit encoded by PPP2CA/PPP2CB), and one of many variable regulatory (B) subunits. The site of phosphoprotein phosphatase (PPP) hydrolysis features a bimetal system (M_1_/M_2_), an associated bridge hydroxide [W^1^(OH^−^)], and a highly-conserved core sequence. In the presumptive common mechanism, the phosphoprotein’s seryl/threonyl phosphate coordinates the M_1_/M_2_ system, W^1^(OH^−^) attacks the central P atom, rupturing the antipodal bond, and simultaneously, a histidine/aspartate tandem protonates the exiting seryl/threonyl alkoxide. Based on studies of PPP5C, a conserved arginine proximal to M_1_ is also expected to bind the substrate’s phosphate group in a bidentate fashion. However, in PP2A isozymes, the role of the arginine (Arg^89^) in hydrolysis is not clear because two independent structures for PP2A(PPP2R5C) and PP2A(PPP2R5D) show that Arg^89^ engages in a weak salt bridge at the B:C interface. These observations raise the question of whether hydrolysis proceeds with or without direct involvement of Arg^89^. The interaction of Arg^89^ with B:Glu^198^ in PP2A(PPP2R5D) is significant because the pathogenic E198K variant of B56δ is associated with irregular protein phosphorylation levels and consequent developmental disorders (Jordan’s Syndrome; OMIM #616355). In this study, we perform quantum-based hybrid [ONIOM(UB3LYP/6-31G(d):UPM7)] calculations on 39-residue models of the PP2A(PPP2R5D)/pSer (phosphoserine) system to estimate activation barriers for hydrolysis in the presence of bidentate Arg^89^-substrate binding and when Arg^89^ is otherwise engaged in the salt-bridge interaction. Our solvation-corrected results yield ΔH^‡^ ≈ ΔE^‡^ = +15.5 kcal/mol for the former case, versus +18.8 kcal/mol for the latter, indicating that bidentate Arg^89^-substrate binding is critical for optimal catalytic function of the enzyme. We speculate that PP2A(PPP2R5D) activity is suppressed by B:Glu^198^ sequestration of C:Arg^89^ under native conditions, whereas the PP2A(PPP2R5D)-holoenzyme containing the E198K variant has a positively-charged lysine in this position that alters normal function.

## 1 Introduction

What is commonly referenced as PP2A (serine/threonine protein phosphatase-2A) in the literature is actually a family of the phosphoprotein phosphatase (PPP) heterotrimeric metalloenzymes composed of a common scaffold (A: PPP2R1), one of many regulatory subunits (e.g., B: PPP2Rn, *n* = 2, 3, 5, 6), and a common catalytic subunit (C: PPP2C) (Janssens et al., 2008; Virshup and Shenolikar, 2009; Brautigan, 2013; Swingle and Honkanen, 2019). A wide array of native species of the PP2A holoenzyme arises from the incorporation of one of two structurally similar isoforms of A, and one of two nearly identical isoforms of C, and one of ∼20 isoforms of B (Jordens et al., 2006; Janssens et al., 2008; Wu et al., 2017). In addition, non-canonical regulatory proteins that do not share structural features of the traditional B-subunits can tether the common A and C subunits into additional unique protein complexes. In total, the PP2A isozymes mediate much of the protein dephosphorylation that occurs in the cell, opposing the actions of kinases in the dynamic regulation of phosphoprotein function (Alberts et al., 1993; Yan et al., 2008; Lee et al., 2010; Wong et al., 2010; Zhou et al., 2017; Ueda et al., 2018; Smith et al., 2019; Papke et al., 2021). Most PP2A isoforms are ubiquitously expressed, with the PPP2C catalytic subunit found at especially high levels in heart and brain tissues. Aberrant PP2A actions have been implicated in several diseases, including cancer, Alzheimer’s, diabetes (Kowluru and Matti, 2012; Sontag and Sontag, 2014; Ruvolo, 2016) and Jordan’s Syndrome (Papke et al., 2021). In addition, genomic mutations in PPP2R5D are recognized causes of intellectual disability (ID) and neurodevelopmental delay disorders (Biswas et al., 2020; Sandal et al., 2021; Oyama et al., 2023). The catalytic behavior of PP2A wherein the regulatory (B) subunit is the B′56δ isoform (encoded by the PPP2R5D gene) is the focus of this work and is denoted here as PP2A(PPP2R5D).

**FIGURE 1:**
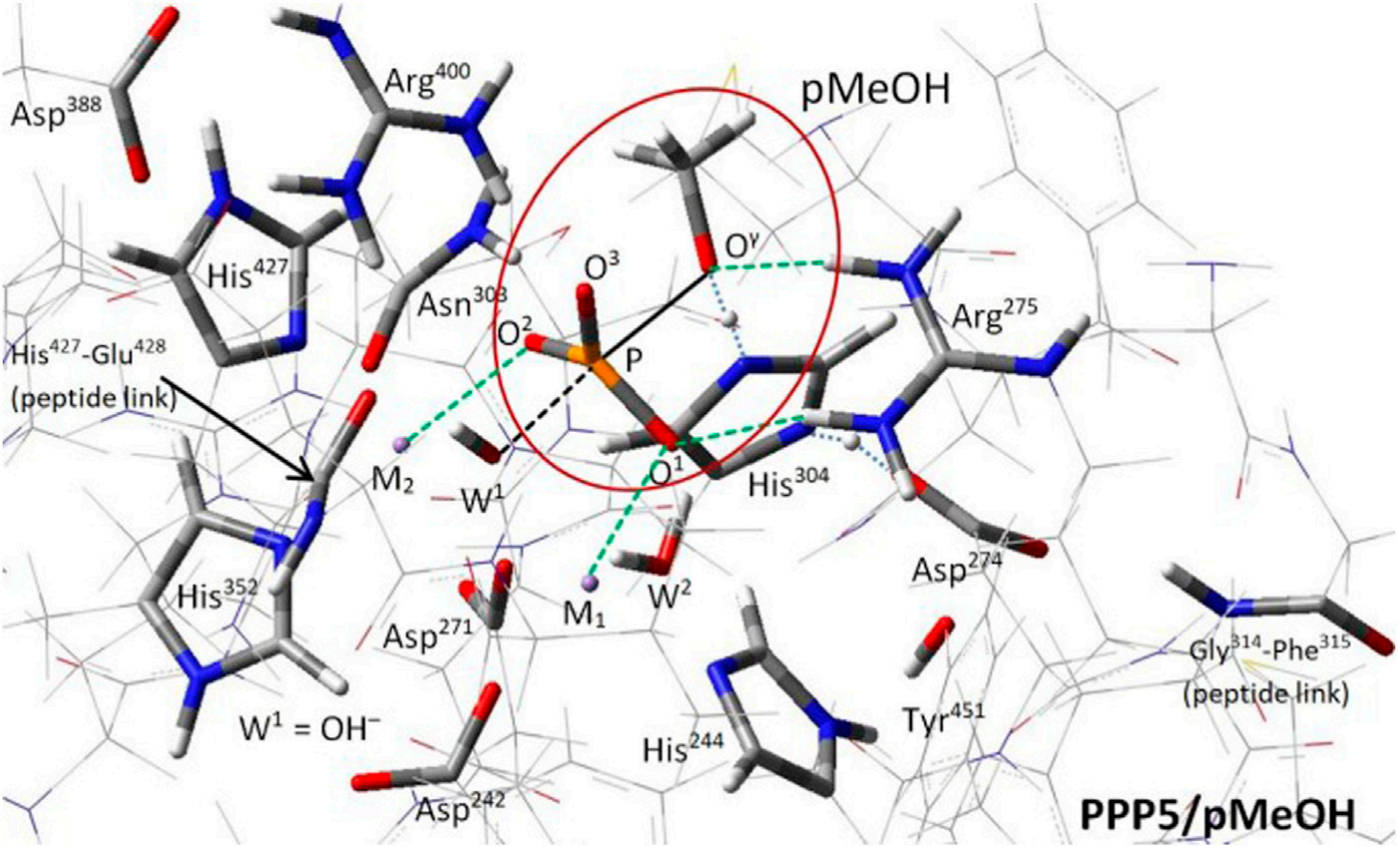
PPP5 catalytic site and transition state structure (Salter et al., 2020). The transition state for hydrolysis of methylphosphate dianion (pMeOH; circled), stand-in for pSer/pThr, is shown. The alignment for backside attack by W^1^(OH^−^) on the P center is indicated by the black dashed/solid lines; the phosphoryl group is bound at O^1^ and O^2^ to M_1_ and M_2_, respectively, and is nearly flat, resulting in a trigonal bipyramidal complex. Arg^275^ has dual H-bonds with O^1^ and O^γ^. Proton transfer from the His^304^/Asp^274^ tandem to O^γ^ (blue dotted lines) to complete the product methanol molecule is concurrent with P center inversion. All labeled residues are conserved across the PPP family, except for those at the two peptide links.

The phosphoprotein phosphatase (PPP) gene family (PPP1C, PPP2C, PPP3C/calcineurin, PPP4C, PPP5C/PP5, PPP6C, and PPPEF/PP7) is a subcategory of the broader class of phosphatases that act on serine and threonine p-sites of proteins (Swingle and Honkanen, 2019). The highly-conserved PPP catalytic core (Huang and Honkanen, 1998) features a bimetal system (M_1_/M_2_; Mn^2+^/Mn^2+^ in PPP2C and PPP5) and a bridge hydroxide W^1^(OH^−^) at the binding site of the phosphate-bearing serine (pSer) or threonine (pThr) of the substrate; also present is a cooperative His/Asp tandem that protonates the exiting seryl/threonyl alkoxide at O^γ^. X-ray diffraction (XRD) structures of PPP5C (Swingle et al., 2004) and PPP1C (Egloff et al., 1995; Goldberg et al., 1995), along with mutation studies (Zhang et al., 1996; Mondragon et al., 1997) and analysis (Egloff et al., 1995; Goldberg et al., 1995; Swingle et al., 2004), established a plausible reaction pathway for generic PPP dephosphorylation wherein a nucleophilic attack on the P center by W^1^(OH^−^) causes inversion as the antipodal P-O^γ^ bond breaks. The mode of binding of the substrate to M_1_/M_2_ and the W^1^(OH^−^) attack were inferred from the aforementioned structures and were later corroborated by small PPP5/pSer cluster calculations by Ribeiro et al. (2013); Figure 1 shows the PPP5 catalytic site from our own recent study of the PPP5/pMeOH (methylphosphate dianion) system in Salter et al. (2020). In that report, we provided computational evidence that the W^1^(OH^−^) attack and protonation of the exiting alkoxide occur in a single concerted step. Further, we asserted that a conserved arginine (PPP5:Arg^275^), located proximal to M_1_, helps to stabilize the transition state (TS) via bidentate binding of the argininium group with the substrate’s ester oxygen (O^γ^) and one of the phosphoryl oxygens (O^1^), Figure 1. The bidentate binding shown in Figure 1 is consistent with, and was initially based upon, the mode of binding evident in PPP5C/phosphate co-crystal (Swingle et al., 2004) and also present in PPP1C/phosphate [pdb entry 4MOV (Choy et al., 2014)] and some PPP3C/phosphate/protein supercomplexes [e.g., pdb entries 2P6B (Li et al., 2007) and 6NUF (Hendus-Altenburger et al., 2019)]. The electron-withdrawing impact of the argininium moiety should make the P center more susceptible to nucleophilic attack; thus, we expect this PPP-conserved arginine to engage in similar substrate binding and TS stabilization in all PPPs, including the PP2A isozymes. For reference, the counterpart of PPP5:Arg^275^ in PP2A’s catalytic subunit (PPP2C) is Arg^89^.

At present, however, there is no direct evidence that PPP5-like Arg^89^-substrate binding occurs in PP2A isozymes denoted as PP2A(PPP2R5X), referring to PP2A species that incorporate any one of the 5 B′ isoforms α to ϵ encoded by the PPP2R5X (X = A-E) genes as the regulatory (B) subunit. In the PP2A(PPP2R5X) isozymes, it is possible that Arg^89^ instead retains a weak salt-bridge interaction with a glutamate residue of the B subunit at the B:C interface rather than engage the substrate’s phosphate group during hydrolysis; the partner glutamate residue in the salt bridge is common to all members of the B′ regulatory class. The Arg^89^-B:Glu^112^ salt bridge is evident in XRD structures of the PP2A(PPP2R5C)/microcystin-LR co-crystal (pdb entries 3FGA (Xu et al., 2009) and 2NYM (Xu et al., 2006); r(N-O^ϵ^) = 3.437 Å and 4.742 Å, respectively), where there is no phosphate moiety bound to the Mn^2+^/Mn^2+^ system. [Note: B:Glu^112^ is residue number 177 in the PP2A-B′ alignment table presented in Figure 3B of Sandal et al. (2021) and is residue number 122 in Figure 3 of Xu et al. (2006)] In these structures, Arg^89^ also interacts with the two carboxylate moieties of microcystin. In a new cryo-EM structure of unliganded PP2A(PPP2R5D), the salt bridge involving B:Glu^198^ may be even stronger, with r(N-O^ϵ^) = 3.219 Å, while Arg^89^ makes additional contacts with the C-terminus of the B subunit, including the hydroxyl group of B:Ser^573^. The significant point is that Arg^89^ cannot engage the substrate’s phosphate moiety as shown in Figure 1 at the same time it is involved in the salt bridge. Also, we note that the backbone position of Arg^89^ as located in the PP2A(PPP2R5X) structures does not quite accommodate PPP5-like bidentate binding of the substrate. Thus, it may be possible that PP2A(PPP2R5X)-mediated hydrolysis proceeds efficiently without direct involvement of Arg^89^. On the other hand, if PPP5-like Arg^89^-substrate binding is indeed required, we can infer that a function of the PPP2R5-encoded subunits might be to alter PP2A(PPP2R5X) catalytic activity by sequestering Arg^89^ via this glutamate under certain conditions. Our motivation for this work is that by understanding of the action of Arg^89^ in wild type PP2A(PPP2R5D), we will be better positioned to uncover why the E198K (Glu^198^ → Lys^198^) single-mutation variant of B′56δ results in irregular protein phosphorylation levels and Jordan’s Syndrome (Houge et al., 2015; Oyama et al., 2023).

**FIGURE 2:**
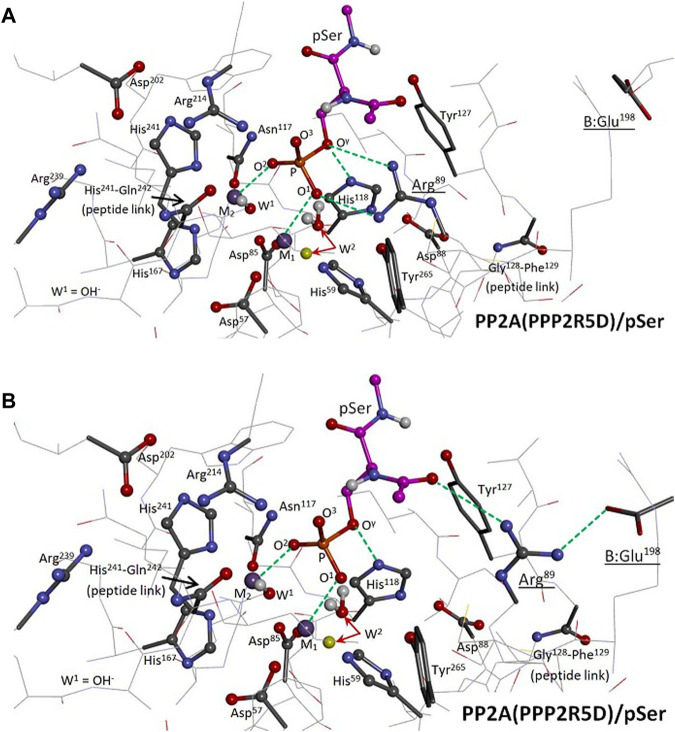
Two-level ONIOM Model of the PP2A(PPP2R5D)/pSer System. The model spans 39 residues; functional groups of the “high-level” region are represented as ball and stick, “low-level” as tube or wire. Selected interactions are represented by green dashed lines. The enzyme’s hydrogen atoms and pSer’s nonpolar hydrogens are hidden from view; the carbon atoms of pSer are in magenta. M_1_ and M_2_ are Mn^2+^ ions. Panel **(A)**: The reactant state of Pathway I is shown, with the phosphate group of pSer bound to both the M_1_/M_2_ system at O^1^ and O^2^ and to Arg^89^ at O^1^ and O^γ^; the coordination of O^γ^ to His^118^ from below is also indicated. The indicated alternative position of W^2^ (yellow) pertains to Pathway I-alt. Panel **(B)**: The reactant state of Pathway II is shown, with Arg^89^ engaged in a salt bridge with B:Glu^198^. The indicated alternative position of W^2^ (yellow) pertains to Pathway II-alt.

To determine if PPP5-like Arg^89^-substrate binding is required for efficient PP2A(PPP2R5D)-mediated hydrolysis, we constructed two versions of a 39-residue model of the catalytic site of the PP2A(PPP2R5D)/pSer system and carried out hybrid quantum-based (QM/QM) calculations, Figure 2. Two cases of Arg^89^ involvement were considered: Pathway I; Figure 2A, is PPP5-like, with bidentate argininium-phosphate binding similar to that of PPP5/pMeOH in Figure 1; in Pathway II; Figure 2B, Arg^89^ is engaged in a salt bridge with B:Glu^198^ and is oriented away from the phosphate moiety of pSer. Closely-related pathways (Pathway I-alt and Pathway II-alt) were explored with W^2^ (the M_1_-bound water) in a secondary position, also indicated in Figure 2. We performed searches for stable reactant and transition states in order to estimate enthalpies of activation (ΔH^‡^ ≈ ΔE^‡^) for these pathways; our best quality estimates were then obtained by calculating solvent-corrected single-point energies.

## 2 Materials and methods

The dianionic form of phosphoserine (pSer) was used throughout our modeling, consistent with kinetic isotope data that indicated that PPPs act on the dianion (Hengge and Martin, 1997) and with the reasoning that a protonated phosphate species would have enhanced acidity when metal bound. The pSer substrate was given acetamide and N-methylamide terminations. Only low-resolution C subunit structures are currently available for PP2A, and no PP2A/phosphate structure has been solved to date. So, in brief, to improve the potential quality of a working QM/QM model, we first refined the best available XRD structure for the C subunit [pdb entry 4I5L (Wlodarchak et al., 2013)] within a B:C dimer assembly using a quantum mechanics/molecular mechanics (QM/MM) hybrid scheme; we then extracted the 39-residue cluster shown in Figure 2 as the basis for a QM/QM model. During the course of our investigation, we found it was necessary to generate two versions of our QM/QM model (Models A and B) in order to fully assess Pathways I, I-alt, II, and II-alt. The details are as follows:

1. Dimer(B′56δ:Cα) construction: The PPP2CA-encoded C subunit (Cα) of the PP2A(R3B) holoenzyme [pdb entry 4I5L (Wlodarchak et al., 2013), 2.43 Å res., residues 3–296] was superimposed to replace the lower-resolution C subunit in a cryo-EM structure of the PP2A(PPP2R5D) holoenzyme (∼3.6 Å res.). The A subunit was subsequently removed, producing a B′56δ:Cα dimer model system. C:Tyr^91^ was found to bump B:Glu^193^ at the B:C interface; so, Tyr^91^ was adjusted to resemble its orientation in the cryo-EM structure to avoid contact. Additionally, residues C:297–302 were spliced in from pdb entry 2IAE (Cho and Xu, 2007) (3.50 Å res.) to fill a sequence gap, and residues C:303–309 were appended from the cryo-EM structure. The Mn^2+^/Mn^2+^ system, associated water, bridge hydroxide, and pSer substrate (ACE-S2P-NME) were inserted; all eight atoms directly coordinated to the Mn^2+^/Mn^2+^ system were set to match that of a small cluster that had been optimized using the B3LYP/6-31G(d) model. C:His^118^ was protonated.
2. Dimer(B′56δ:Cα)/pSer MM optimization: Two versions of the dimer/pSer system were created at this juncture: For Model A, the sidechain of Arg^89^ was reoriented from its XRD position [pdb entry 4I5L (Wlodarchak et al., 2013)] to guide binding to the pSer phosphate group as in Pathway I; and for Model B, Arg^89^ was left in its XRD position. The dimer/pSer systems were solvated in a TIP3P water box with an 8 Å buffer; Na^+^ and Cl^−^ ions were added to neutralize and then to approximate 0.15 M NaCl. The systems were optimized by the Amber 18 SANDER program (Case et al., 2018) through 10,000 steps, using ff14SB force field parameters under periodic boundary conditions and a 10 Å cutoff. For the bridge hydroxide, gaff o and ho parameters were assigned to the O and H atoms, with −1.205 and +0.205 charges, respectively. During these optimizations, the bimetal system and the eight directly-coordinated atoms were held fixed.
3. Dimer(B′56δ:Cα)/pSer QM/MM optimization: The two versions of the solvated dimer/pSer system were reoptimized under AMBER’s QM/MM hybrid scheme of Walker et al. (2008), using PM7 for the quantum model under periodic boundary conditions with cut = 8 Å. To stabilize PM7 convergence, the quantum region (total charge = +1) was expanded in 6 optimization stages (100 steps or rms Force <0.1 kcal mol^−1^Å^−1^; 66 and 56 steps in the final stages, respectively) to ultimately incorporate pSer, the Mn^2+^/Mn^2+^ system, and 39 selected residues of the catalytic site, including B:Glu^198^. During these optimizations, the metal ions and the eight coordinated atoms were held fixed. Note: AMBER automatically imposed aldehyde and neutral amine terminations at breaks in the peptide backbone at the QM/MM boundary.
4. ONIOM QM/QM calculations: The quantum regions from Step 3 were extracted as Models A and B to perform calculations using Gaussian16 (Frisch et al., 2016). ONIOM(UB3LYP/6-31G(d):UPM7) (Ditchfield, 1971; Hehre, 1972; Becke, 1993; Lee et al., 1998; Rassolov et al., 1998; Dapprich et al., 1999) partial optimizations for stable structures and searches for TS structures were carried out as in Salter et al. (2020). Atoms were selected for the “high-level” and “low-level” regions as indicated in Figure 2. The atoms of pSer, the M_1_/M_2_ system, W^1^(OH^−^), and W^2^ were allow to move, along with the sidechains of the following residues: Arg^89^, Arg^214^, Asp^202^, Asn^117^, Tyr^265^, Tyr^127^, His^118^, Asp^88^, plus the amide H atom at the 128–129 peptide link, totaling 137 free atoms. As the Mn^2+^/Mn^2+^ electronic system is an antiferromagnetic singlet state (5 “up”/5 “down”), the proper “high-level” B3LYP wavefunction was attained by first constructing an appropriate spin-unrestricted guess wavefunction. The “low-level” PM7 states could not be manipulated in the same manner; instead, we converged to stable spin-unrestricted open-shell singlets (1 “up”/1 “down”) using STABLE = opt. PM7 convergence was aided by using the quadratic convergence option (SCF = yqc). For Pathway II geometry searches, a brief, localized pre-optimization was performed to allow the formation of the salt bridge between Arg^89^ and B:Glu^198^ before refreezing the position of B:Glu^198^. Also, when the alternative position for W^2^ was explored in Pathway I-alt and Pathway II-alt calculations, W^2^ was set by a brief, localized pre-optimization of W^2^. Some TS searches were initially aided by temporarily freezing selected internal coordinates by using GEOM = addgic. Calculations were performed using INT = grid = superfine. Searches were judged complete when the RMS force met the default target, as satisfying the full set of default convergence criteria was not practical for a system of this size. Analytic vibrational frequencies were computed to confirm local curvature of the energy surface. Single-point calculations using the default continuum solvation model (SCRF = solvent = water, sas) were used to estimate solvent-corrected energies.

## 3 Results

Activation barriers for the hydrolysis of pSer in the catalytic site of PP2A(PPP2R5D) are presented in Table 1 along with selected geometric parameters; Table 1 summarizes the results of ONIOM QM/QM calculations performed on two versions our 39-residue model: one in which Arg^89^ was guided to bidentate binding of the pSer substrate while permitting backbone relaxation during model construction (Model A), and one in which Arg^89^ was left in its original XRD position (Model B). Superposition shows that the binding in Model A induced a relative shift compared to Model B in the position of C^α^:Arg^89^ of about 0.75 Å. Meaningful results for Pathway I and I-alt were achieved only with use of Model A; that is, our attempts to achieve bidentate Arg^89^-pSer binding using Model B were not successful, as the aforementioned structural relaxation is evidently required to suitably position Arg^89^. Vibrational frequency analysis confirmed all reactant and product geometries as local minima and assigned exactly one imaginary frequency to TS structures. Coordinates of the optimized structures and animations of the imaginary modes are available in the Supplementary Materials.

**TABLE 1:** Activation barriers and selected geometric parameters for PP2A(PPP2R5D)/pSer hydrolysis^a^.

	Reactant state		Transition state			ΔH^‡^ (kcal/mol)^b^	
	Inversion dihedral^c^	r(P-O^γ^) (Å)	Inversion dihedral^c^	r(P-O^γ^) (Å)	r(P-O^W1^) (Å)	Gas phase	Solvent corrected
Pathway I: Model A	−23.7°	1.711	+10.6°	2.492	1.889	+24.3	+15.5
Pathway I-alt: Model A^d^	−24.7°	1.713	+8.9°	2.427	1.928	+24.5	+16.4
Pathway II-alt: Model B^d^	−25.3°	1.700	0.0°	2.191	2.141	+24.8	+18.8
Pathway II-alt: Model A^d^	−25.8°	1.702	+5.0°	2.074	2.014	+26.1	+22.3
PPP5/pMeOH^d^	−21.1°	1.759	+4.1°	2.223	1.950	+8.6	+10.0

^a^ ONIOM(UB3LYP/6-31G(d):UPM7) model systems and atom labeling are shown in Figure 2.

^b^ ΔH^‡^ ≈ ΔE^‡^.

^c^ Inversion dihedral given by d(O^2^,O^1^,O^3^, P). More positive (or less negative) values favor the product side of the inversion process; the dihedral for bound HPO_4_^2−^is ∼ +21°.

^d^ The “alt” designation refers to the alternative position taken by W^2^ indicated in Figure 2.

^e^ pMeOH, methyl phosphate dianion. Data from Salter et al. (2020).

**FIGURE 3:**
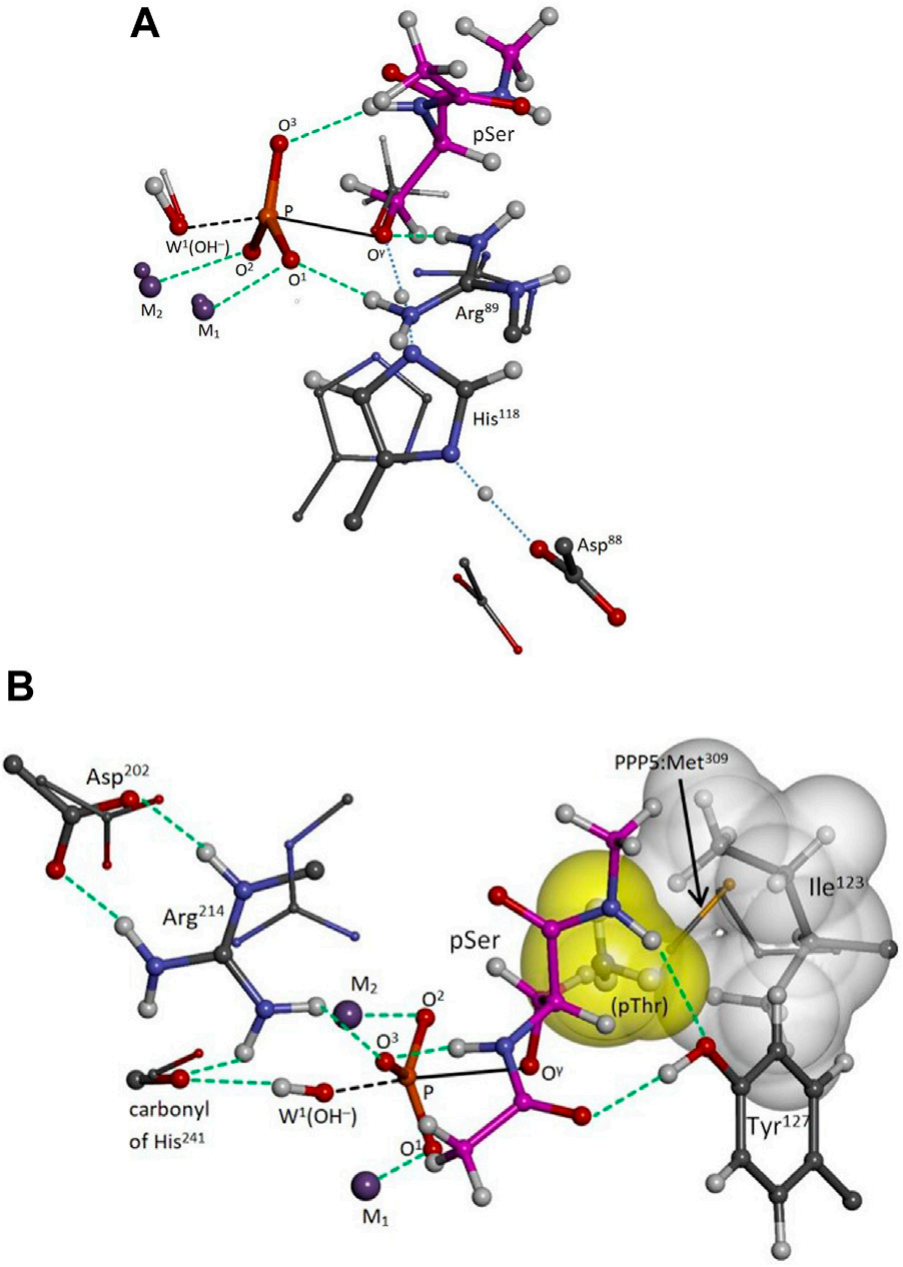
PP2A(PPP2R5D/pSer transition state. Selected moieties of the PPP5/pMeOH system are superimposed as small balls and sticks; carbon atoms of pSer are in magenta; selected interactions are represented by green dashed lines. The distorted trigonal bipyramidal complex is indicated as black dashed/solid lines. Panel **(A)**: View of Arg^89^-pSer bidentate binding at O^1^ and O^γ^ and of the protons of the His^118^/Asp^88^ tandem in transit (blue dotted lines). The His/Asp tandem is shifted relative to its PPP5 counterparts. The phosphoryl moiety favors the product side of the reaction slightly more than in PPP5. Panel **(B)**: The tyrosyl group of Tyr^127^ anchors pSer with two H-bonds at backbone sites; pSer’s amide hydrogen also registers internally with O^3^. The anticipated pose of pThr is also indicated, with its γ-methyl (yellow surface) occupying a free space capped by the sidechain of Ile^123^ (white surface), a space sometimes partially occupied in PPP5 by the terminal methyl of the homologous PPP5:Met^309^ as shown. Arg^214^ is shifted relative to its PPP5 counterpart and interacts with the backbone carbonyl of His^241^ as well as with O^3^.

Pathways I and I-alt, investigated using Model A, exhibit bidentate Arg^89^-pSer binding at O^1^ and O^γ^ in the reactant, transition, and product states. With W^2^ positioned as in PPP5, Pathway I has a barrier of ΔH^‡^ = +15.5 kcal/mol (solvent-corrected), Table 1, and the reaction enthalpy is mildly positive (ΔH ≈ ΔE = +6.0 kcal/mol), and it is the energetically-preferred pathway for PP2A(PPP2R5D)-mediated hydrolysis. The impact of solvent on the energetics is significant, as evinced by the 8.8 kcal/mol drop in the barrier from the gas-phase value of +24.3 kcal/mol. Occupation of the W^2^ secondary position gives rise to a barrier of +16.4 kcal/mol in the very similar Pathway I-alt. The TS for Pathway I is more advanced toward the product geometry in terms of P-O^γ^ and P-O^W1^ bond distances and the inversion dihedral than in comparison with the TSs for Pathways I-alt, II, or II-alt. Also, in the reactant state, the P-O^γ^ bond is more elongated than in Pathway II or II-alt. The TS is illustrated in Figure 3, where some features are compared to the PPP5/pMeOH system; Figure 3A shows the similarity in the trigonal bipyramidal TS complex to that of PPP5/pMeOH and highlights the difference in the relative position of the His/Asp tandem. The histidine planes of the two systems roughly coincide, but N^ϵ^ of His^118^ is shifted by 0.75 Å and the carboxylate group of Asp^88^ is displaced by about 2 Å. Figure 3B indicates the H-bonding role of Tyr^127^ in positioning pSer, which was not known about previously for PPP-mediated hydrolysis. Additionally, the alignment of the Asp^202^/Arg^239^/Gln^212^ grouping that directs Arg^214^ is shifted relative to the homologous PPP5 grouping. As a result, C^ζ^ of Arg^214^ is shifted by 1.72 Å relative to its PPP5 counterpart and Arg^214^ has a short-range interaction with the backbone carbonyl of His^241^, as well as with O^3^.

Animation of Pathway I’s imaginary mode (441.7*i* cm^-1^) confirms that the TS is concerted, just as in PPP5/pMeOH (Salter et al., 2020); that is, W^1^(OH^−^) attacks the P center at the same time that the exiting seryl alkoxide is protonated by His^118^. This TS mode differs, however, in that the inversion motion of the P center is less pronounced relative to the motion of H^+^ transfer from His^118^, presumably because the inversion is already well advanced at 10.6° versus 4.1° for PPP5/pMeOH. Also, the mode shows little movement of Tyr^265^, which would be expected to aid the breaking of the Arg^89^-O^1^ interaction in the product state, and as already stated, the bidentate motif persists in the Pathway I product state. In contrast, the mode in Salter et al. (2020) indicates movement that eventually releases Arg^275^ from its interaction with O^1^ of the HPO_4_^2-^ product, as Tyr^451^ follows it.

During optimizations and TS searches for Pathway II, W^2^ dissociates from M_1_ to engage instead with the adjacent Tyr^265^ when starting from the PPP5 position. Instead, we were able to find the aforementioned alternative position for W^2^ located farther from Tyr^265^, Figure 2B, to investigate the energetics of Pathway II-alt. Using Model B, Pathway II-alt has a solvent-corrected barrier of ΔH^‡^ = +18.8 kcal/mol, which is higher than that of Pathway I by 21%; without solvent correction, Pathway I is more favorable by 0.5 kcal/mol. Not surprisingly, when Pathway II-alt was investigated using Model A, which was built for optimization for Arg^89^-pSer bidentate binding, an even higher barrier value (+22.3 kcal/mol) was obtained. We note that Arg^89^, while engaged in the salt-bridge with B:Glu^198^, is not completely isolated from pSer in Pathway II-alt; Arg^89^ interacts with the carbonyl at the acetamide end of pSer as indicated in Figure 2B.

## 4 Discussion

Arg^89^ of the shared PP2A catalytic subunit, and its counterpart in the other PPPs, is a conserved, mobile residue known to take different positions depending upon the ligand present in the co-crystal. Mobility of this arginine is essential, allowing entry and exit by substrate and products. Interestingly, PP2A(PPP2R5D)’s Arg^89^ can engage in a weak salt-bridge interaction at the interface between the catalytic and regulatory domains of the holoenzyme according to experimental PP2A(PPP2R5C) and PP2A(PPP2R5D) structures. We have considered the question as to whether hydrolysis can proceed in PP2A(PPP2R5X) isozymes without direct interaction between Arg^89^ and the substrate’s phosphate moiety. Using a quantum-based computational model of the PP2A(PPP2R5D)/pSer system prepared from structural data, we have found that when Arg^89^ is engaged with Glu^198^ in a salt bridge (Pathway II-alt), the energy of activation is higher than when bidentate Arg^89^-pSer binding occurs (Pathway I), Table 1. Geometric features of the reactant and transition states for Pathway I also support the conclusion that the bidentate motif, established for PPP5 in our earlier study (Salter et al., 2020), is needed for optimal efficiency of PP2A(PPP2R5X) hydrolysis and is likely a universal characteristic of the PPPs.

For PPP5, we reported that post-transition state, as the product alcohol recedes, Arg^275^ turns to maintain the O^γ^ interaction, while the O^1^ interaction ends; Arg^275^ is isolated from the HPO_4_^2-^ product as Tyr^451^ follows the movement of Arg^275^ (Salter et al., 2020). Here, in our Pathway I results for PP2A(PPP2R5D), the Arg^89^-HPO_4_^2-^ interaction at O^1^ is maintained in the product state. We believe the former behavior would be present in a fully relaxed, high-resolution PP2A(R5X) model, wherein Tyr^265^, which is located in the flexible β12-β13 loop, is induced into the proper position to perform its role. We would expect the relative shift in the position of the His/Asp tandem (Figure 3A) to be less dramatic as well. Nevertheless, these differences may account in part for the higher activation barrier we obtain for PP2A(PPP2R5D/pSer (+15.5 kcal/mol) as compared to the PPP5/pMeOH value (+10.0 kcal/mol) determined in Salter et al. (2020).

Our modeling has revealed additional elements of generic substrate binding in the PPPs. We have assigned the Tyr^127^ a role in substrate orientation and binding through dual H-bonding at the substrate’s backbone carbonyl and amide hydrogen, Figure 3B. Tyrosine at this position is nearly conserved among the PPPs, with the lone exception of phenylalanine in PPP3C. This discovery was beyond the scope of our previous PPP5 modeling with pMeOH as substrate in Salter et al. (2020) and the scope of the small PPP5 cluster model in Ribeiro et al. (2013), which did not include the homologous PPP5:Tyr^313^. These anchoring interactions should now aid us in projecting the binding orientations of tripeptide-sized substrates. Of course, modification of Tyr^127^ in PP2A would directly impact substrate binding, and if, as assays now indicate, Tyr^127^ is a p-site (unpublished data), phosphorylation at that position would likely cause loss of catalytic activity. It is perhaps worth noting that the Y127C variant of PPP2CA leads to very low PP2A activity, though the activity loss is attributed to the linked loss of methylation at Leu^309^—a modification apparently needed to enhance B:C affinity (Reynhout et al., 2019). Also, we have projected a plausible position for pThr’s γ-methyl as indicated in Figure 3B, where the methyl group fills a space capped by Ile^123^. As Ile^123^ is not conserved among the PPPs, this site may be responsible for some selectivity of pThr versus pSer as substrate. The equivalent residue is methionine in PPP5 and PPP7, leucine in PPP3C, and isoleucine for the remaining PPPs. Occupation of the space by Met^309^ in PPP5 varies. For example, in the PPP5/phosphate co-crystal [pdb entry 1S95 (Swingle et al., 2004)], PPP5:Met^309^ is turned away to leave the space vacant, but partially occupies the space in PPP5/endothall [pdb entry 3H61 (Bertini et al., 2009)] and in the similar co-crystal [pdb entry 4ZX2 (Chattopadhyay et al., 2016)] upon which our PPP5/pMeOH cluster model in Salter et al. (2020) was based.

Our main conclusion is that while in the apparent native state (with a weak salt bridge between Arg^89^ and the B′-conserved glutamate) the PP2A(R5X) isozymes are not optimally active until Arg^89^ is released to permit bidentate Arg^89^-substrate binding. We propose that a function of the B′ regulatory subunits might be to downregulate activity by sequestering Arg^89^ via the salt bridge until something else is altered, perhaps the states of the p-sites located at the catalytic subunit’s C-terminus, or until a specific amino acid sequence of the protein substrate is recognized at the enzyme surface. Our tentative interpretation regarding the B′56δ-E198K variant is that the resulting PP2A(PPP2R5D) holoenzyme is incapable of sequestering Arg^89^ in this manner, resulting in the loss of Arg^89^-mediated regulation of catalytic activity and serious health consequences (Houge et al., 2015; Oyama et al., 2023).

## Data Availability

The raw data supporting the conclusion of this article will be made available by the authors, without undue reservation.
